# Flexible assertive community treatment (FACT) model in specialist psychosis teams: an evaluation

**DOI:** 10.1192/pb.bp.116.053967

**Published:** 2017-08

**Authors:** Loopinder Sood, Andy Owen, Richard Onyon, Aarohi Sharma, Jessica Nigriello, Dominic Markham, Hannah Seabrook

**Affiliations:** 1 Coventry & Warwickshire Partnership NHS Trust, Stratford-upon-Avon, UK; 2 University Hospital Coventry and Warwickshire, Coventry, UK

## Abstract

**Aims and method** The impact of flexible assertive community treatment (FACT) has been observed in people previously supported by assertive community treatment (ACT) teams, but its effect on those previously with a community mental health team (CMHT) has not been studied in the UK. An observational study was conducted of 380 people from 3 CMHTs and 95 people from an ACT team, all with a history of psychosis, following service reconfiguration to 3 FACT teams.

**Results** People previously with a CMHT required less time in hospital when the FACT model was introduced. A smaller reduction was observed in people coming from the ACT team. Both groups required less crisis resolution home treatment (CRHT) team input.

**Clinical implications** FACT may be a better model than standard CMHT care for people with a history of psychosis, as a result of reduced need for acute (CRHT and in-patient) services.

Recent years have seen a widespread disinvestment from assertive community treatment (ACT), with many teams being merged into generic community mental health teams (CMHTs).^1,2^ Such changes have been criticised for moving away from evidence-based systems, instead adopting untested models of service delivery, with poorer quality care, described in a King's Fund report as ‘a leap in the dark’.^3^ In some cases new services have adopted a flexible ACT (FACT) model, with people able to access intensive support delivered in the community using a team case-load and ACT principles, as and when they require it.^4,5^ In this model, care coordinators manage individual case-loads, but also work together to provide shared care for people at times of increased need, enabling seamless transition between high- and low-intensity care.

An observational study in the UK has not shown negative effects in people previously supported by ACT teams in terms of hospital admissions or need for crisis resolution home treatment team (CRHT) interventions.^6,7^ However, there have been no UK studies assessing whether there could be advantages for people previously supported by a CMHT but now receiving FACT. It might be expected that some people who have not previously had access to periods of more intensive support within the CMHT could benefit from the FACT approach, perhaps with a reduced need to have interventions from a CRHT or hospital admission.

## Method

In South Warwickshire there had previously been a single, well-established ACT team, with outcome data over a 10-year period showing a reduced need for time in hospital.^8^ The area was also served by three generic CMHTs and a single early intervention team. The early intervention services were maintained, whereas each of the three CMHTs was divided into a team for people with a history of psychosis (recovery teams) and a team for people with other non-organic mental disorders. At the same time they became ‘age-independent’, with no upper age limit. The previous ACT team was disbanded and merged into the new recovery teams, which were configured to deliver services using the FACT model. A few months prior to the changes, the ACT team had absorbed a community rehabilitation team and therefore fidelity to the original ACT model had reduced. Characteristics of the ACT, CMHT and FACT teams are shown in Table 1.

**Table 1:** Characteristics of the different teams

	CMHT	ACT	FACT
Case mix	People with psychotic or non-psychotic disorders	People with history of severe psychosis who have difficulty engaging with traditional services and often with comorbid problems	People with a history of psychosis

Age range, years	17–65 for new referrals, no upper age limit for existing users of the service	17–65 for new referrals, no upper age limit for existing users of the service	17 and upwards for new and existing users of the service

Care planning	Individual case management	Shared care	Individual case management with periods of shared care as needed

Interface with mental health professionals	Referrals made between professionals when needed	All professionals involved in delivery of care without referrals, on a needs-led basis	All professionals involved in delivery of care without referrals, on a needs-led basis

Care coordinator: patient ratio	30	12–15	25

ACT, assertive community treatment; CMHT, community mental health team; FACT, flexible assertive community treatment.

There was no change in acute hospital bed availability during the study period. However, a long-stay rehabilitation ward was closed at about the same time as the other changes took place, with most residents being discharged either to nursing homes or to intensive community placements with 24-hour live-in staff support. This group had been care-coordinated by the ACT team both prior to and following discharge.

The current study was a service evaluation of the new FACT-based recovery teams. It assessed their impact in enabling people to avoid time in hospital, to reduce the use of crisis home treatment support, and to examine how much face-to-face support people received from the new service. There were multiple changes associated with setting up the teams, all of which took place in June 2014. Many people experienced a change in care coordinator and/or consultant, and there were various teething problems with the transition. In order to avoid these becoming confounding variables, we chose to study a 13-month period starting 6 months after the creation of the new services: December 2014 until January 2016. We compared this with a 13-month period in the old services a year earlier (December 2012 until January 2014).

The trust uses a computerised notes system for all staff in the community, which constitutes the sole record of any contacts with clients. It can generate detailed reports on clinical contacts between specified time periods, broken down by team or staff member, and is routinely used for gathering trust performance data. Because people are constantly moving in and out of services, we decided to study only those people who were open to the new FACT service during the 13-month study period, and who had also been in one of the 3 CMHTs or the ACT team during the comparison 13-month period. Because of the closure of the rehabilitation ward, there was potential for a considerable impact on bed use data in the ACT arm of the study – the patients, having spent several years in hospital, were moving to nursing care or 24-hour live-in support. For this reason, we excluded from the bed use analysis those who were being discharged from hospital after several years into nursing or live-in community care.

## Results

A total of 475 people who had also been with one of the previous legacy teams the year before were identified as being open to the new service. Of these, 95 had previously been with the ACT team and 380 with one of the CMHTs. Results were analysed separately for these two groups. Tests of significance between the old systems and the new FACT service were carried out using 2-tailed paired *t*-tests or, when data were skewed, using the Wilcoxon signed rank test. A Monte Carlo permutation test, as described by Good^9^ and derived from Fisher,^10^ was used when there was no standard statistical method available, such as to compare partially paired data. This type of testing gives a *P*-value directly (much like Fisher's exact test) without an intermediary test statistic such as a *t*-value. To keep the false detection rate (i.e. the overall type 1 error) low at 0.05 on account of multiple testing, we used the Benjamini-Hochberg^11^ correction, which gave a significance level alpha of 0.0288. This means that *P*-values of less than 0.0288 are significant. Where a significant difference was observed in one group but not in the other, *post hoc* power calculations were carried out in order to check for any potential type 2 errors. Demographic and clinical characteristics of people from the two legacy teams are shown in Table 2.

**Table 2:** Demographic and clinical characteristics of the cohorts

Previous team	ACT (*n* = 95)	CMHT (*n* = 380)
Gender, male: %	66.0	54.2

Age, years: mean	45.3	47.7

Time in services, years: mean	13.7	11.0

ICD-10 diagnosis, %		
Schizophrenia	78.3	53.4
Schizoaffective disorder	16.3	4.7
Bipolar affective disorder	4.3	28.2
Other	1.1	13.8

ACT, assertive community treatment; CMHT, community mental health team.

### Face-to-face contacts with the FACT teams

For people previously with the ACT team, the number of face-to-face contacts with a member of the new FACT team reduced from 1.16 to 0.69 per week, with a corresponding reduction in mean duration of contacts from 65 to 38 minutes per person. These differences were statistically significant and are of similar magnitude to the changes observed in the other UK study of FACT.^6,7^ The number of contacts by support workers was not significantly different (0.25 compared with 0.29), but the proportion increased from 22 to 43%. In other words, the reduction of face-to-face contacts in the new FACT system for people previously in the ACT team was a result of less involvement of qualified staff. The number of community-based contacts reduced significantly in the new service, but the proportion was greater, indicating that, overall, more contacts had been lost in clinic settings compared with those in the community. For people previously with a CMHT there was very little difference in number and duration of contacts when the service adopted the FACT model. However, there was greater use of support workers and more contacts were in community settings, consistent with the principles of the FACT model (Table 3).

**Table 3:** Contacts with FACT team compared with previous service (ACT or CMHT)

Previous team	ACT (*n* = 95)			CMHT (*n* = 380)		
	ACT	FACT	*P*	CMHT	FACT	*P*
Face-to-face contacts per week: mean	1.16	0.69	<0.0001^a^	0.47	0.45	0.6018^a^
Duration, minutes: mean	64.80	38.13	<0.0001^a^	26.38	25.33	0.5544^a^
By support worker: mean	0.25	0.29	0.3941^a^	0.07	0.15	<0.0001^a^
By support worker: %	21.45	42.60	<0.0001^b^	14.18	33.86	<0.0001^b^
In the community: mean	0.74	0.51	0.0001^a^	0.25	0.29	0.0314^a^
In the community: %	63.81	73.73	0.0001^b^	52.42	63.85	<0.0001^b^

CRHT use						
People with any face-to-face contact: *n* (mean)	28 (0.29)	16 (0.17)	0.0023^a^	128 (0.34)	88 (0.23)	<0.0001^a^
Face-to-face contacts: mean	5.83	1.94	0.0237^a^	7.14	2.83	<0.0001^a^
Duration of face-to-face contacts per person, minutes: mean	151.87	51.03	0.0455^a^	250.98	97.15	<0.0001^a^
People with any telephone or face-to-face contact: *n* (mean)	29 (0.31)	23 (0.24)	0.1584^a^	134 (0.35)	119 (0.31)	0.0190^a^

Hospital use						
Days in hospital: mean	31.76	25.86	0.7413^c^	19.34	12.35	0.0006^c^
Admissions: mean	0.20	0.12	0.0776^c^	0.25	0.18	0.0535^c^
People with any admission: *n*	15	11	0.3458^b^	71	52	0.0388^b^

ACT, assertive community treatment; CMHT, community mental health team; FACT, flexible assertive community treatment.

a. 2-tailed paired *t*-test.

b. Monte Carlo permutation test.

c. 2-tailed Wilcoxon.

*P*>0.0288 not significant (after Benjamini–Hochberg correction).

### Contact with the CRHT and hospital use

The number of face-to-face contacts with the CRHT was compared before and after the changes, and significant reductions were seen in both groups. Similarly, the number of people who required any face-to-face support from the CRHT was significantly lower following the changes.

For people who had previously been with the ACT team there was a 19% reduction in number of days spent in hospital, which failed to reach statistical significance. However, the power calculated *post hoc* was only 4%, which indicates that the numbers were insufficient to conclude there was no difference following the change of model. There was also a reduction in mean number of admissions in this group but numbers were too low for a meaningful comparison to be made. In the CMHT group, reductions in bed use were much greater, with a 36% reduction following the introduction of the FACT model, which reached statistical significance. There was also a non-significant reduction in admissions in this group.

## Discussion

### People previously with a CMHT

There have been no other UK studies exploring the effect of the FACT model on people who had previously been with a CMHT. We observed that these people experienced less than half the number of face-to-face interventions with the CRHT than when they were with a CMHT, which was statistically significant. This is consistent with the FACT philosophy of enabling people to seamlessly move to a high-intensity team approach at times of increased need.^12^ Hence, it is possible that during periods of crisis, people were able to receive intensive community support within the FACT team, reducing the need for transfer to the CRHT. Similarly, the reductions in bed use would be consistent with the ability of the FACT model to support people at times of crisis with less need for admission. There were no changes in background bed availability in the services that would provide an alternative explanation for these reductions.

### People previously with the ACT team

The other UK evaluation of FACT considered 112 people who had previously been with an ACT service, comparing their hospital and CRHT use before and after the change,^6,7^ but without an appraisal of the impact of people going to FACT from a CMHT. Our findings for people who had previously been supported by the ACT team were similar, with no evidence of adverse consequences in terms of increased need for admission or increased crisis home treatment team contact in the first year. This was despite a considerable reduction in face-to-face support from mental health services. In fact, bed use was reduced, but not significantly, although this has to be interpreted with caution and may not be clinically meaningful as the number of people admitted during the study period was very low.

A possible explanation for this is that a FACT approach could be a more efficient model than ACT because people only receive high-intensity team-based interventions at times of need, freeing up resources for those who most need them. However, our previous follow-up study of the ACT population in South Warwickshire^8^ showed that most people, once they had been with the service for 5 years, reverted to a relatively low level of bed use. The average time with the ACT team had been over 6 years, and by the time the services changed most of these people were relatively stable. Hence, it might be expected that they would cope well with a move to a less intensive service. Any conclusion that there was no evidence of harm when moving from ACT to FACT would therefore be limited to the context of people who have already received a period of several years of intensive ACT interventions.

### Limitations

Because there were a number of changes to services, including moving to an ‘age-independent’ model, caution needs to be exercised in interpreting the findings as being solely attributable to the FACT model. One of the limitations of the observational design is the possibility of regression to the mean or background variations which could contribute to reduced hospital use or less contact with the CRHT. Change point analysis can mitigate against this,^13–15^ particularly if combined with start points staggered in time in order to reduce the effect of wider system changes which might influence results. However, because the time period under study was relatively short and the changes in team structure occurred on the same date it was not possible to use this technique. The most robust method for addressing confounding factors would be a randomised controlled trial, but this was beyond the scope of our pragmatic evaluation. The pragmatic method was limited to routinely collected contact data and did not capture more personally meaningful information about satisfaction, social functioning and engagement with services, which are known benefits of ACT.^16^

ACT teams have had varying levels of success in terms of achieving fidelity to the model.^17^ Without the use of an objective measure, such as the Dartmouth Assertive Community Treatment Scale (DACTS),^18^ it is not possible to know with certainty the degree to which the South Warwickshire team was practising according to the ACT principles. Although previous DACTS measures taken several years earlier had shown high fidelity, this had been eroded with less use of shared case-loads and lower staff to patient ratios. Hence, the observations about the outcome for people who had been with the ACT team cannot be extrapolated with certainty to other ACT teams with higher fidelity. This argument also applies to the Firn studies^6,7^ of dismantling ACT teams, which failed to measure ACT fidelity. Although a FACT fidelity scale is available (from The Netherlands),^12^ this has never been validated in a UK setting. As Dutch FACT teams also undertake the role of a 24-hour crisis home treatment service, it would not be meaningful to use this scale with a service in the UK, where this function is provided by separate teams.

### Conclusions

This is the first study in the UK which has examined the impact of adopting the FACT model on people previously supported by a CMHT within a generic community psychosis service. Although limited by the observational design, the results are consistent with the hypothesis that FACT may be of benefit to this group, who previously did not have access to ACT. People who had been with the CMHT were able to receive increased support delivered with a team case-load at times of increased need, a key component of the FACT approach, thereby reducing their need for the help of the CRHT. We would argue that there is still a case for maintaining ACT teams, which have been much more rigorously assessed than FACT, and that the benefits to patients justify the investment in these services. However, where mental health providers are planning to disband ACT services, there would be value in configuring new teams according to the FACT model, which appears to be a safe alternative in the short term for people who have been with an ACT team for several years.
